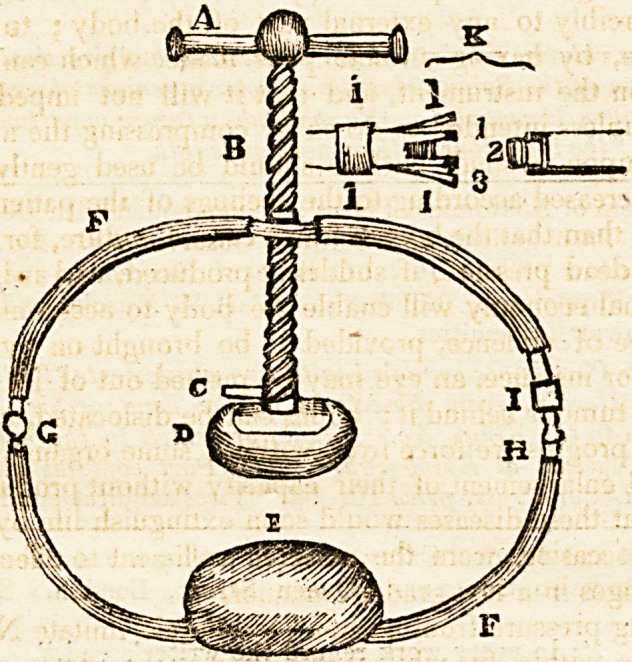# Extra-Limites

**Published:** 1824-09-01

**Authors:** 


### EXTRA-LIMITES.

### I.

***A Description of an Instrument for Applying Local Pressure, without Interfering with the General Circulation. Its Use in a Case of Ovarian Dropsy.*** **By H. SEARLE, Surgeon, London.**

THE above Instrument is recommended for the purpose of applying pressure to any part of the human frame, without including within its influence, parts which are not required to be pressed, (excepting the portion which must necessarily sustain the counter-pressure.) The construction of it is more economical than any other that has hitherto been used, which may be seen by referring to the Figure. A. the sliding handle; B. the screw; C. a pin, by which the compressing pad D. is unscrewed, in order to substitute another of a different size or shape; E. the pad for counter-pressure; F F. the elastic steel ring, covered with leather; G. its joint, which allows it to be applied with great expedition; H. the lock shut; I. its slide; K. the lock opened, which is formed of two slips L L. split from their centre-piece, and have a pin at each extremity, 1. 2. 3. Its three portions having a bore in each, which together, equal the length of the two pins, and when received in fastening, of course, meet in the bore of the middle portion, 2.

In order to open this lock, draw back the slide I, and the two slips being made to diverge will draw the two pins out of the middle portion, and thus liberate it. To shut it, bring the ends of the ring together, and draw the slide home to the lock. This lock was contrived by Mr. Botschan, Instrument Maker.

Sir Astley Cooper constructed an instrument on a similar principle, about twenty years ago, to compress a popliteal aneurism; the counter-pressure was thrown upon the knee and trochanter major, and the screw was held by a semicircular piece of iron. I have not seen the description of it. It is to be found in the 8th vol. of the Med. Phys. Journal. The patient could endure the application of it only for a few hours; it being a dead pressure, and at once applied with severity. Its further use was abandoned.

The advantages of the present apparatus are, that it may be applied mildly or forcibly to any external part of the body; to a large or a small surface, by having suitable pads made, which can be easily exchanged upon the instrument, and that it will not impede the general circulation, unless intended to do so by compressing the arterial trunk.

Pressure upon a small surface should be used gently at first, and gradually encreased according to the feelings of the patient, for nothing is more true, than that the human frame cannot endure, for any length of time, severe dead pressure, if suddenly produced, and it is well known that the animal economy will enable the body to accommodate itself to a great degree of violence, provided it be brought on by an insensible increase. For instance, an eye may be pushed out of its socket by the growth of a tumour behind it; bones can be dislocated, curved or perforated, by a progressive force from within; some organs can sustain an hundred-fold enlargement of their capacity without producing the least pain, and that these diseases would soon extinguish life by the irritation they would occasion, were the pressure sufficient to effect these extraordinary changes in a less gradual manner.

In applying pressure from without, we should imitate Nature in producing it from within, for while it becomes progressively augmented in any one direction, there are both a yielding and a resistance in the opposite, so that a slight undulation attends the whole process, as if through the agency of a spring. It is therefore recommended, that the ring of this instrument be made of steel sufficiently thin and tempered, to allow of a continual play by virtue of its elasticity, and the relief this will afford to the sufferings of the patient, was proved in the following case of ovarian dropsy.

A young lady had been troubled with this complaint several years, and various modes of treatment had been tried by the different medical gentlemen she had consulted. She then became my patient, and after prescribing some medicine, which had no better effect, I recommended her to try pressure, which was accomplished by placing two books upon the abdomen strapped down by two leathern belts well wadded. This somewhat lessened the ovarial tumor, but the legs became swelled, and their skin much inflamed. This plan was persevered in during a few months with great distress, and she was prevented taking proper exercise. Finding the immediate effect of this plan advantageous to the complaint, which had now existed upwards of six years, I advised her to be tapped, that it might be attempted under more favourable circumstances. Sir A. Cooper performed the operation, the patient being in a recumbent position, which is unquestionably preferable to that of sitting, and he took great pains in expressing all the water, which was about 16 pints, and contained in one cyst. I then returned to the use of pressure, and several towels were placed upon the abdomen, and bound on with about forty yards of calico and flannel, four inches wide, upon these a book or two were strapped with the two leathern belts as before. But this pressure was so steady and general upon the abdominal vicera, that digestion became completely stopped, and the hips excoriated from the bandages. These sufferings were endured with fortitude for a week, when the whole was undone, and there appeared to be a re-accumulation of about three pints; as this quantity of fluid was much less than is usual a week after tapping, she was bound up again, not quite so high nor so tightly, and small bolsters were placed so as to protect the hips. Her diet was as much animal food, with brandy and water, as she could conveniently take to lessen the disposition to serous effusion. This mode was persevered in for several weeks longer, when the effects upon the hips and ribs, rendered some other method of applying the pressure indispensible. Her health in the mean time had considerably improved, for symptoms of a typhoid character, during the first few days, had been produced.

A steel plate, six inches by six, was then fitted to the back, and well padded; another, a front plate, was made seven inches in length, and oval, that it might fall within the ilia and pubes. At each side of the back plate, a semicircular spring was attached, which came forward, and was connected with a screw made to act upon the centre of the front plate; the circle of apparatus being complete; it answered extremely well for a day or two; after which, the back became excessively painful, and required further protection. Therefore, two pads, seven inches by three each, and one in thickness, stuffed with horsehair, were stitched together like the lids of a book, so as to form a joint which enabled them, when inclined a little towards each other, at an obtuse angle round the back, to throw the counter pressure upon the soft parts on each side of the spine, while the lower parts rested upon the sacrum. This has been worn ever since, without any inconvenience, which is principally owing to the springs. It can be easily put on; it should then be screwed down till the pulsation of the aorta be apparent, and one turn of the screw backwards, adjusts it to the degree of pressure, which has been borne twelve months without any symptom of a return of the complaint. The abdominal parietes not yet having contracted to their natural state, she cannot leave off the instrument. No attempt has been made to prove whether the disease would return, without its use. The patient does not present any outward appearance of her wearing it. It may be observed, that the viscera are so exceedingly mobile, that no adhesions can have taken place.

Sir A. Cooper has lately recommended a leathern belt, for stopping the further progress of the complaint previous to tapping; whether he has ever tried it, I cannot say, but, in this instance, the little good afforded, was far exceeded by the inconvenience of swelled legs.

There are many cases in which this description of instrument might be found useful. It can be easily modified in its construction, and be adapted to any part of the body. It is certainly deserving another trial in aneurism. The obliteration of an artery, probably, would not be a very tedious process, could the apparatus, by means of its spring, and judicious management, be borne, as the anastomosing vessels soon enlarge, and divert the circulation of the blood from the portion rendered impervious, and which, being in a diseased state, would the more readily have its coats become inflamed. It might require two degrees of pressure; one immediately above the aneurismal sac, to keep the blood out of it, and the other upon it, milder, to excite inflammation. Experiment would soon prove whether this regulation would be necessary. Pressure might also be applied to the chest or back, without impeding respiration; to the carotid artery, when required to be taken up; or to any part which was circumscribed.

---

### II.

### Dr. GORDON SMITH'S QUERIES.

We are requested, on the part of Dr. Gordon Smith, to state, that he is under the necessity of postponing his intention of lecturing on Political Medicine, this season. His friends are aware how frequently he has, of late, been laid aside; and his health is yet in too precarious a state to warrant the contraction of so impotant an engagement with the Public. He trusts that the delay which may intervene, before he can execute an object he has so much at heart, will prove advantageous to the ultimate accomplishment of his wishes.

In the mean time, he has resolved to occupy himself, more fully than has yet been done by any individual, with the important and ill-understood subject, of proving or disproving the vitality of new-born children—or, to speak technically, of Infanticide. There is no point in forensic medicine, on which so much perplexity exists, and for the elucidation of which so little has, in reality, been done. Dr. Smith is persuaded, that this is owing, in great measure, to want of due pains fairly bestowed—for, whether the existing state of knowledge be estimated positively, or negatively, no one who has at all looked at the question, can be unaware that there is much work to be gone through, before satisfactory conclusions can be formed.

It is almost impossible for any individual—at least, Dr. S. feels that it is beyond the compass of his own unaided power, to do justice to the problem; as few who possess the means, can be supposed to have the leisure requisite to collect, arrange, and apply, the necessary data. But he relies on the zeal and liberality of the profession, to assist him in so important an undertaking. He, therefore, embraces this opportunity of soliciting, from those who may have opportunities of handling the bodies of new-born infants, a few items of information, which he hopes will not occasion much trouble, and the aggregate of which, he trusts, he may be enabled to apply with precision and advantage—so that the truth may be established (either one way or other, and to its proper amount) with regard to certain doctrines that have been bandied about for many years, without any satisfactory estimate as to their fair practical import.

Simple answers to the following queries, form the object of the present application—premising that the subjects chosen must be *perfect ;* that is, of ordinary development, free from redundant parts, mutilation, disease, putrefaction, &c. and such as are unquestionably of the class to which they may be assigned ; if, in any particular instance, there should be points in morbid anatomy, which, in certain cases, might greatly assist in coming to appropriate conclusions, they will require to be carefully stated.

The subjects, being classed, *first,* as STILL-BORN—or such *as have never respired ;* and, *secondly,* as VIVI-BORN, or those that *have been born alive,* and have respired, but died within twelve hours. Dr. Smith begs to submit the queries in the following manner.

Class I. STILL-BORN *Subjects*—Required the

1. Sex ?—2. Period of gestation when born ?—3. How long *known,* or *supposed* (as the case may be) to have been dead *in utero ?*—4. The cause of death ?—5. Nature of the labour ?—6. Exterior aspect of the body ?—as to α Colour; β Integrity; γ Development; δ Formation; ε Marks of violence, ecchymosis, &c. or any *peculiarity* —7. Length from the crown of the head to the under surface of the heel ?—8. Point at which the middle length of the body falls, to be given, as regards its distance, from the umbilicus ?—9. Weight of the whole body, prior to any interference with its integrity; to be accurately given in ounces and fractions, stating the species of weight used ?—10. Aspect of the lungs, *in situ,* on laying open the thorax; and form of the diaphragm ?—11. Weight of the lungs, separated from all attachments—avoiding the spilling of contents ?—12. Weight of any fluid that may escape from the trachea, on holding the lungs in an inverted position (without squeezing them) and the fluid to be described ?—13. Result of placing the lungs in a washing basin of water, first entire, then separately; —*i. e.* the right and left lung each by itself—noting if there be any difference of buoyancy in either, and which—as, also, any morbid appearances that may present themselves in these organs ?—14. Weight of the liver, &c. managed in a similar way ?—15. State of the alimentary canal, with regard to contents ?—16. State of the urinary bladder ?—17. State of the gall bladder ?—18. State of the ductus, arteriosus, and venosus ?—19. Colour and consistence of the blood, expressing the part or parts of the body in which the observation may have been made.

Class II. VIVI-BORN SUBJECTS. Required the

1. Sex ?—2. Period of gestation when born ?—3. The first actions. α As to crying, or manner in which respiration was manifested ? — β State of the umbilical cord ?—γ As to evacuations per anum and urethram ?—4 Cause, manner, and time of death ? Then to follow the queries, as in the other case.

It is not expected or desired that any individual shall take the trouble to furnish *a list.* A single case properly investigated, and clearly stated by an intelligent hand, will be worth hundreds such as seem to have been collected abroad, one hardly knows how. In order, however, to impart necessary satisfaction as to the authenticity of the materials, it will be essential, that those who may be pleased to transmit the results of their enquiries, should verify them with their signatures ; and, in all cases, when practicable, Dr. Smith will scrupulously acknowledge his obligations.

Communications may be forwarded for Dr. Gordon Smith, to the care of Messrs. Underwood, 32, Fleet Street, London ; and he leaves the *economy* of transmission entirely to the convenience and discretion of correspondents.

### MR. BATTLEY ON THE COMPONENTS OF OPIUM.

*To the Editors of the Medico-Chirurgical Review.*

GENTLEMEN,

In your last number, I stated that I had subjected twenty-six pounds of opium to the action of water, and that a residuum or refuse of three pounds was left in deposit. I showed, also, that the morphium of opium (so called) was contained or included in this residuum.

Finding much inconvenience from the attempt to continue my experiments upon the large scale of twenty-six pounds, I have proceeded upon eight pounds only, and to that scale or standard, the following statements must be referred. I do not, however, find the same *proportional* results, and I apprehend that equality, in this respect, is not to be expected from any two quantities of opium, although of equal weight.

Eight pounds (avoirdupois) of opium, when *perfectly* dried, weighed about seven pounds, and imparted to distilled water 4 lbs. 12 oz. leaving a residuum of 2 lbs. 4 oz. when dried; the latter containing, as I continue to assert, the *morphium.* This residuum, subjected to the process described in my last paper, produced of pure crystals, 8 drachms 44 grains.

The 4 lbs. 12 oz. imparted to the distilled water, when dried, was subjected successively, four times, to the action of cold water, and precipitated 12 oz. 60 grains. This precipitate dried, and then macerated in dilute acetic acid and ammonia in excess, yielded,

| | drachms. | grains. |
|---|---|---|
| *Morphium* - - - - - - - - - - - - - - - - - - - - - | 2 | 4 |
| Pure resinous matter - - - - - - - - - - - - - - - - | 3 | 40 |
| Remained in the filter - - - - - - - - - - - - - - - | 0 | 14 |
| | 5 | 58 |

Leaving 9 oz. 26 grains not acted upon, and the remainder suspended in the maceration.

Little, if any, effect, followed from the immersion of the 9 oz. 26 grains in four pounds of alcohol *(cold)* during fourteen hours :—when heated to boiling temperature, the alcohol became deeply tinged, and the boiling was repeated in fresh alcohol, eight to ten times, until the alcohol ceased to be affected. The following are the results of this operation, viz.

| | oz. | drachms. | grains. |
|---|---|---|---|
| Pure resin - - - - - - - - - - - - - - - - - - - - - - | 4 | 2 | |
| Not acted upon - - - - - - - - - - - - - - - - - - | 4 | 6 | 40 |

One moiety of the latter, immersed in a mixture of,

| | |
|---|---|
| Distilled water - - - - - - - - - - - - - - - - - - - - | 2 pints. |
| Ammonia - - - - - - - - - - - - - - - - - - - - - - - | 1 oz. |

left in deposit matter of a grey slaty appearance, weighing, when dried, 1 oz. 2 drachms, 20 grains, and imparted to the fluid, the same weight of 1 oz. 2 drachms, 20 grains, resembling, in appearance, hard extract of liquorice.

The other moiety was immersed in diluted nitric acid, and remained in a temperature of 100°, during several days, when a mass was formed, which imparted to distilled water, 2 drachms, 10 grains, of a bright deep yellow colour, (when condensed) in quality adhesive, and to the taste bitter,—acrid. Of the remainder, 1 drachm, 40 grains, boiled in alcohol, yielded to that menstruum, 22 grains of a dingy yellow appearance, and of the taste of raw coffee.

The 4 lbs. 12 oz. (reduced by the precipitation before-mentioned, of 12 oz. 60 grains) in the state of extract, had entirely lost its characteristic properties of taste and smell, and had become simply bitter to the taste, but intense in degree, and of an agreeable odour, and upon being alternately extended and relaxed by the hand, altered from a dark dull appearance to a bright yellow colour.

Of this mass:—

Four ounces were diffused in ten pints of distilled water; the mixture, turbid, upon filtering became transparent, and the test paper showed the presence of an *acid*. To this clear or transparent solution, was added, one pint of acetic acid, and after twenty-four hours, ammonia was added in excess; a precipitation ensued, which, when washed and dried, weighed twenty-one grains, of a dark, shining, brittle quality, and pulverized readily. Boiling alcohol dissolved 19 grains, leaving a refuse of two grains. Upon recovering the extract, (19 grains) from the alcohol, not a crystal was formed, *thus shewing the entire absence of morphium*, from the mass from which the four ounces were taken.

Four ounces diffused in the same quantity of distilled water, produced a mixture slightly turbid, which became perfectly clear upon passing the filter, showed an acid as before, and upon adding liq. potass. so long as the presence of acid was indicated by the test paper, the solution became exceedingly turbid, and deposited a substance, which, when washed and dried, weighed three drachms; this substance yielded to boiling alcohol (frequently repeated) crystals, 2 drachms, 33 grains, and left on the filter 21 grains saline particles.

Four ounces diffused in the same quantity of distilled water, presented similar effects, until, by the addition of ammonia, instead of liq. potass. a considerable deposition, of a yellow colour and globular formation was produced, weighing, when dried, about one ounce.—Severe illness prevented the further prosecution of this branch of the investigation.

To four ounces diffused in like manner, magnesia was added; the deposition weighed 1 ounce, 2 quarters, 1 drachm, and yielded to boiling alcohol, still more frequently repeated than before, crystals 3 drachms, 21 grains.

I have now brought this enquiry to a point which will enable me, in a future paper, to state what separations from opium are effected upon obtaining the liq. op. sedativ. and *what those separations severally are*, and I shall also endeavour, in the same paper, to show the constituents of that preparation.

I am, GENTLEMEN,

Your obedient Servant,

RICHARD BATTLEY.

FORE STREET, 14th August, 1824.

## Figures and Tables

**Figure f1:**